# Propofol Protects Against Focal Cerebral Ischemia via Inhibition of Microglia-Mediated Proinflammatory Cytokines in a Rat Model of Experimental Stroke

**DOI:** 10.1371/journal.pone.0082729

**Published:** 2013-12-09

**Authors:** Rong Zhou, Zailiang Yang, Xurong Tang, Yan Tan, Xiaofeng Wu, Feng Liu

**Affiliations:** 1 Department of Operating Room, Children’s Hospital, Chongqing Medical University, Chongqing, China; 2 Ministry of Education Key Laboratory of Child Development and Disorders, Children’s Hospital, Chongqing Medical University, Chongqing, China; 3 Hematopoietic Stem Cell Transplantation and Gene Therapy Center, Affiliated Hospital of Academy of Military Medical Sciences, Beijing, China; 4 State Key Laboratory of Trauma, Burn and Combined Injury, Daping Hospital, Third Military Medical University, Chongqing, China; 5 Research Institute of Surgery, Daping Hospital, Third Military Medical University, Chongqing, China; 6 Department of Anesthesiology, Children’s Hospital, Chongqing Medical University, Chongqing, China; University of Colorado Denver, United States of America

## Abstract

Ischemic stroke induces microglial activation and release of proinflammatory cytokines, contributing to the expansion of brain injury and poor clinical outcome. Propofol has been shown to ameliorate neuronal injury in a number of experimental studies, but the precise mechanisms involved in its neuroprotective effects remain unclear. We tested the hypothesis that propofol confers neuroprotection against focal ischemia by inhibiting microglia-mediated inflammatory response in a rat model of ischemic stroke. Sprague-Dawley rats were subjected to middle cerebral artery occlusion (MCAO) for 2 h followed by 24 h of reperfusion. Propofol (50 mg/kg/h) or vehicle was infused intravenously at the onset of reperfusion for 30 minutes. In vehicle-treated rats, MCAO resulted in significant cerebral infarction, higher neurological deficit scores and decreased time on the rotarod compared with sham-operated rats. Propofol treatment reduced infarct volume and improved the neurological functions. In addition, molecular studies demonstrated that mRNA expression of microglial marker Cd68 and Emr1 was significantly increased, and mRNA and protein expressions of proinflammatory cytokines tumor necrosis factor-α, interleukin-1β and interleukin-6 were augmented in the peri-infarct cortical regions of vehicle-treated rats 24 h after MCAO. Immunohistochemical study revealed that number of total microglia and proportion of activated microglia in the peri-infarct cortical regions were markedly elevated. All of these findings were ameliorated in propofol-treated rats. Furthermore, vehicle-treated rats had higher plasma levels of interleukin-6 and C-reactive protein 24 h after MCAO, which were decreased after treatment with propofol. These results suggest that propofol protects against focal cerebral ischemia via inhibition of microglia-mediated proinflammatory cytokines. Propofol may be a promising therapeutic agent for the treatment of ischemic stroke and other neurodegenerative diseases associated with microglial activation.

## Introduction

Stroke is the leading cause of death and the most frequent cause of long-term disability in the adult population worldwide [1]. Ischemic strokes are the most common type of stroke, representing about 87% of all strokes [2]. Cerebral ischemia induces acute inflammation by triggering excessive production of proinflammatory cytokines in the brain as well as in peripheral blood, which exacerbate brain damage and are related to poor clinical outcome in patients with ischemic stroke [3]. 

Microglia are major immune cells in the central nervous system, which are activated rapidly in response to brain injury [4] or during neurodegenerative processes and produce proinflammatory cytokines, growth factors, reactive oxygen species, nitric oxide, and glutamate [5,6]. Although activation of microglia is necessary and crucial for host defense, the over-activation of microglia results in deleterious and neurotoxic consequences. Experimental studies have shown that resident microglia in the brain are activated within minutes of ischemia onset and release multiple proinflammatory cytokines, such as tumor necrosis factor-α (TNF-α), interleukin-1β (IL-1β) and interleukin-6 (IL-6), which play a crucial role in the progression of neuronal loss and brain injury following ischemic stroke [7-9]. Thus, development of agents that reduce microglial activation in the brain and inhibit the release of proinflammatory cytokines is considered to be an important therapeutic strategy for ischemic stroke. 

Propofol (2,6-diisopropylphenol) is an intravenous hypnotic agent widely used for induction and maintenance of anesthesia during surgeries. In addition, Propofol has antiinflammatory properties, reducing production of proinflammatory cytokines, altering expression of nitric oxide, and inhibiting neutrophil function [10]. An *in vitro* study recently showed that propofol almost completely inhibits lipopolysaccharide-induced activation of microglia and the production of proinflammatory cytokines [11]. A number of experimental studies have reported that propofol ameliorates neuronal injury in animal models of ischemic stroke [12-14]. However, the precise mechanisms involved in its neuroprotective effects remain unclear. In this study, we tested the hypothesis that propofol attenuates cerebral ischemic injury by inhibiting microglia-mediated inflammatory response in a rat model of ischemic stroke. 

## Methods

### Animals

Male Sprague-Dawley rats weighing 250-300 g were purchased from Beijing Laboratory Animal Research Center (Beijing, China). Animals were housed and cared for in the Animal Resource Center and allowed free access to food and water. All procedures were reviewed and approved by the Institutional Animal Care and Use Committee at the Chongqing Medical University and were performed in accordance with the “Guiding Principles for Research Involving Animals and Human Beings”. 

### Experimental protocol

The animals were randomly assigned to 3 groups (n=20 for each group) as follows: (1) middle cerebral artery occlusion (MCAO) group treated with propofol (MCAO+PRO). Rats were subjected to MCAO for 2 h followed by 24 h of reperfusion and infused intravenously with propofol (50 mg/kg/h) using syringe pump at the onset of reperfusion for 30 minutes; (2) MCAO group treated with vehicle (saline) (MCAO+VEH). Same as group (1), but these rats were infused intravenously with saline at the onset of reperfusion for 30 minutes; (3) sham-operated group (SHAM). Rats were subjected to sham MCAO without treatment. The dose for intravenous infusion of propofol was derived from a previous study in which such dose of propofol significantly reduced infarct size 24 h after MCAO in rats [13]. Based on a formula for dose translation from animal to human [15], a dose of 50 mg/kg/h of propofol in rats is roughly equivalent to a dose of 8.1 mg/kg/h in human, which is within the infusion rates of propofol for clinical use in human. At the end of the protocol (24 h after MCAO and reperfusion), neurological deficit scores and motor coordination were evaluated. Rats were then sacrificed, the blood samples were collected for biochemical measurements and the brains were removed for infarct volume assessment, molecular analysis or immunohistochemical study. 

### Induction of MCAO

Transient MCAO was induced by the intraluminal suture method as previously described [16-18]. Briefly, rats were anesthetized with an intraperitoneal (i.p.) injection of pentobarbital sodium (50 mg/kg). Body core temperature was maintained within a normothermic range (37°C to 38°C) with a temperature-controlled heating pad. A 4/0 surgical nylon monofilament with a silicone-beaded tip was introduced into the right internal carotid artery through the external carotid artery to occlude the origin of the middle cerebral artery. After 2 h of occlusion, the monofilament was removed to allow reperfusion for 24 h. In addition, the left femoral artery was cannulated for monitoring blood pressure (BP) and heart rate (HR) and for arterial blood gas measurements. The left femoral vein was cannulated for the administration of drugs. BP and HR were continuously recorded on a computer using the PowerLab software (PowerLab/8SP, Chart 5.0; ADInstruments Pty, Ltd., Castle Hill, Australia). Blood gas measurements were performed 15 min after the onset of ischemia or reperfusion using a blood gas analyzer (Compact 3, AVL Medizintechnik).

### Assessment of neurological outcome

Eight rats from each group were used for assessment of neurological outcome. Neurological deficit scores were evaluated 24 h after ischemia using an eight-point scale as described previously [17]. The score was 0 for no apparent deficits; 1 for failure to extend left forepaw fully; 2 for decreased grip of the left forelimb; 3 for spontaneous movement in all directions, contralateral circling only if pulled by the tail; 4 for circling or walking to the left; 5 for walking only if stimulated; 6 for unresponsiveness to stimulation and with depressed level of consciousness; and 7 for death.

Measurement of motor coordination was performed 24 h before and after ischemia, respectively. The experimental procedure was described previously [19]. Briefly, the time that the rats stayed on a rotating rod was recorded automatically in each case for up to 3 minutes. The trial was conducted five times for each rat, and the mean riding time was used as the mean value for this test. When the time of riding was over 3 minutes, the rat was released from the rod, and the riding time was recorded as 3 minutes. 

At the end of the observation period, these rats were euthanized with an overdose of anesthesia and brains were quickly removed for assessment of infarct volume, as previously described [20]. Briefly, brains were sectioned at 2-mm intervals throughout the rostrocaudal axis of the striatum. Slices were then staining with 2% 2,3,5 triphenyltetrazolium chloride (TTC) for 15 min at 37°C. Slice images were digitalized and infarct areas were analyzed using NIH Image 1.60. The Complete lack of staining with TTC was defined as the infarct lesion. The infarct volume was expressed as a percentage of the contralateral hemisphere. 

### Real-time PCR analysis

The mRNA expression of microglial markers (CD68 and Emr1) and proinflammatory cytokines (TNF-α, IL-1β and IL-6) in the peri-infarct cortical tissue was measured with real-time PCR. Rats (n=8 for each group) were euthanized 24 h after MCAO, and the brains were removed and cut into seven serial 2-mm-thick coronal sections. The peri-infarct cortical tissue was dissected from the coronal brain sections for extraction of total RNA and protein using an operating microscope as described previously [21]. The total RNA was extracted using TRI Reagent (Molecular Research Center, Inc) and reverse transcribed into cDNA. mRNA levels for CD68, Emr1, TNF-α, IL-1β, IL-6 and GAPDH were measured with SYBR green real-time PCR. The sequences for primers used were summarized in Table **1**. Real-time PCR was performed using the ABI prism 7000 Sequence Detection System (Applied Biosystems, Carlsbad, CA). The values were normalized to GAPDH and expressed as a fold change relative to the SHAM group.

**Table 1:** Primer sequences for real-time PCR.

**Primer name**	**Forward primer (5’→3’)**	**Reverse primer (5’→3’)**
CD68	CTTCCCACAAGCAGCACAG	AATGATGAGAGGCAGCAAGAGA
Emr1	AATCGCTGCTGGCTGAATACGG	CCAGGCAAGGAGGGCAGAGTT
TNF-α	GAGAGATTGGCTGCTGGAAC	TGGAGACCATGATGACCGTA
IL-1β	CCTCTGCCAAGTCAGGTCTC	GAATGTGCCACGGTTTTCTT
IL-6	CACAAGTCCGGAGAGGAGAC	CAGAATTGCCATTGCACAAC

### Western blot analysis

The protein levels of proinflammatory cytokines TNF-α, IL-1β and IL-6 in the peri-infarct cortical tissue were measured by Western blot. The peri-infarct cortical tissue was dissected from the coronal brain sections and homogenized in lysis buffer. The protein concentration in the supernatant was measured with the BCA protein assay Kit (Pierce, Rockford, IL, USA). Equivalent amounts of protein were separated on 12% SDS-polyacrylamide gels and transferred to polyvinylidene difluoride membranes (Millipore Corporation, Bedford, MA, USA). The membranes were blocked with 3% nonfat dry milk and then incubated using primary antibody to TNF-α, IL-1β, IL-6 and β-actin (Santa Cruz Biotechnology Inc, Santa Cruz, CA) at 4°C overnight. After three washing, the membranes were incubated with horseradish peroxidase-conjugated second antibody (Santa Cruz Biotechnology Inc, Santa Cruz, CA) for 1 h at room temperature. The signal was visualized using the enhanced chemiluminescence (ECL) detection system (Amersham) and the densities of the immunobands were quantitated. All data were corrected and normalized to β-actin.

### Immunohistochemistry

Twenty-four hours after MCAO, four rats from each group were perfused transcardially with heparinized saline followed by ice-cold 4% paraformaldehyde in phosphate buffered saline. Brains were removed and fixed overnight in 4% paraformaldehyde at 4°C and then immersed in 30% sucrose. Brain tissue was sliced into 20-μm serial coronal sections using a cryostat. Standard immunohistochemical procedures were performed according to a previous study [22]. Briefly, the sections were blocked by 0.5% H_2_O_2_ for 30 min and then incubated in 10% normal horse serum for 60 min to facilitate antibody penetration. Subsequently, the sections were incubated for 72 h with a mouse monoclonal primary antibody directed against CD11b (clone OX-42) (1:100, Chemicon, Temecula, USA) in 2% normal horse serum and 0.2% Triton X-100 in phosphate buffered saline. This was followed by incubations in a biotinylated antimouse secondary antibody raised in horse (1:100, Vector Laboratories, Burlingame, USA) for 2 h. The sections were exposed to DAB reagent (Vector Laboratories, Burlingame, CA), counter-stained with hematoxylin, dehydrated in ethanol, cleared with xylene, and coverslipped with mounting medium. 

Morphological analysis and quantification of microglia were performed with a light microscope as described [22]. Non-activated microglia were distinguished by their small soma from which there emanated extensive, highly branched, long, thin processes, a morphology termed ramified. Activated microglia were defined by the following criteria: stronger immunohistochemical staining for the marker CD11b (clone OX-42), the presence of a clearly enlarged soma and marked changes in the appearance of the processes which were now reduced in number, but considerably thicker and shorter giving a stubby appearance. The number of activated and non-activated microglia was counted in several 0.2 × 0.2 mm squares and the average was calculated.

### Biochemical assays

Blood samples were collected 24 h after MCAO for measurements of plasma proinflammatory cytokines (TNF-α, IL-1β, IL-6 and C-reactive protein) by ELISA kits (Biosource International Inc, Camarillo, CA or R&D Systems Inc, Minneapolis, MN).

### Statistical analysis

Data are expressed as mean±SEM. The significance of differences in mean values was analyzed by one-way or two-way repeated-measure ANOVA followed by Fisher's *post hoc* test. *P*<0.05 was considered statistically significant.

## Results

### Hemodynamic and physiological variables

To eliminate potential confounding factors on neurological outcomes, hemodynamic and physiological variables, including BP, HR and arterial blood gases, were monitored and controlled before, during and after MCAO. As shown in Table **2** and **3**, no significant differences among groups in mean BP, HR, arterial pH, carbon dioxide tension (Pco_2_) and arterial oxygen tension (Po_2_) were observed at each time point before, during MCAO and during reperfusion.

**Table 2:** Systemic hemodynamic variables during MCAO and reperfusion.

	**Pre-Ischemia**	**Ischemia 15 min**	**Ischemia 35 min**	**Reperfusion 5 min**	**Reperfusion 35 min**
*MBP (mmHg)*					
SHAM	115 ± 12	116 ± 8	115± 12	115 ± 9	114 ± 11
MCAO+VEH	113 ± 9	110 ± 10	112 ± 13	109 ± 12	109 ± 10
MCAO+PRO	115 ± 11	117 ± 10	113 ± 9	110 ± 11	112 ± 15
*HR (beats/min)*					
SHAM	342 ± 15	346 ± 16	345 ± 19	345 ± 13	343 ± 16
MCAO+VEH	346 ± 13	349 ± 12	344 ± 12	343 ± 15	340 ± 11
MCAO+PRO	340 ± 18	342 ± 17	341± 15	342 ± 17	340 ± 15

**Table 3:** Physiological variables during MCAO and reperfusion.

	**Ischemia**			**Reperfusion**		
	***pH***	***PaO_2_ (mmHg)***	***PaCO_2_ (mmHg)***	***pH***	***PaO_2_ (mmHg)***	***PaCO_2_ (mmHg)***
SHAM	7.36 ± 0.04	134 ± 15	38 ± 5	7.39 ± 0.06	133 ± 14	37 ± 7
MCAO+VEH	7.41 ± 0.04	131 ± 17	37 ± 3	7.37 ± 0.09	136 ± 12	36 ± 8
MCAO+PRO	7.39 ± 0.03	135 ± 12	40 ± 6	7.41 ± 0.03	139 ± 11	37 ± 5

### Propofol ameliorated MCAO-induced neuronal injury

**Figure 1:**
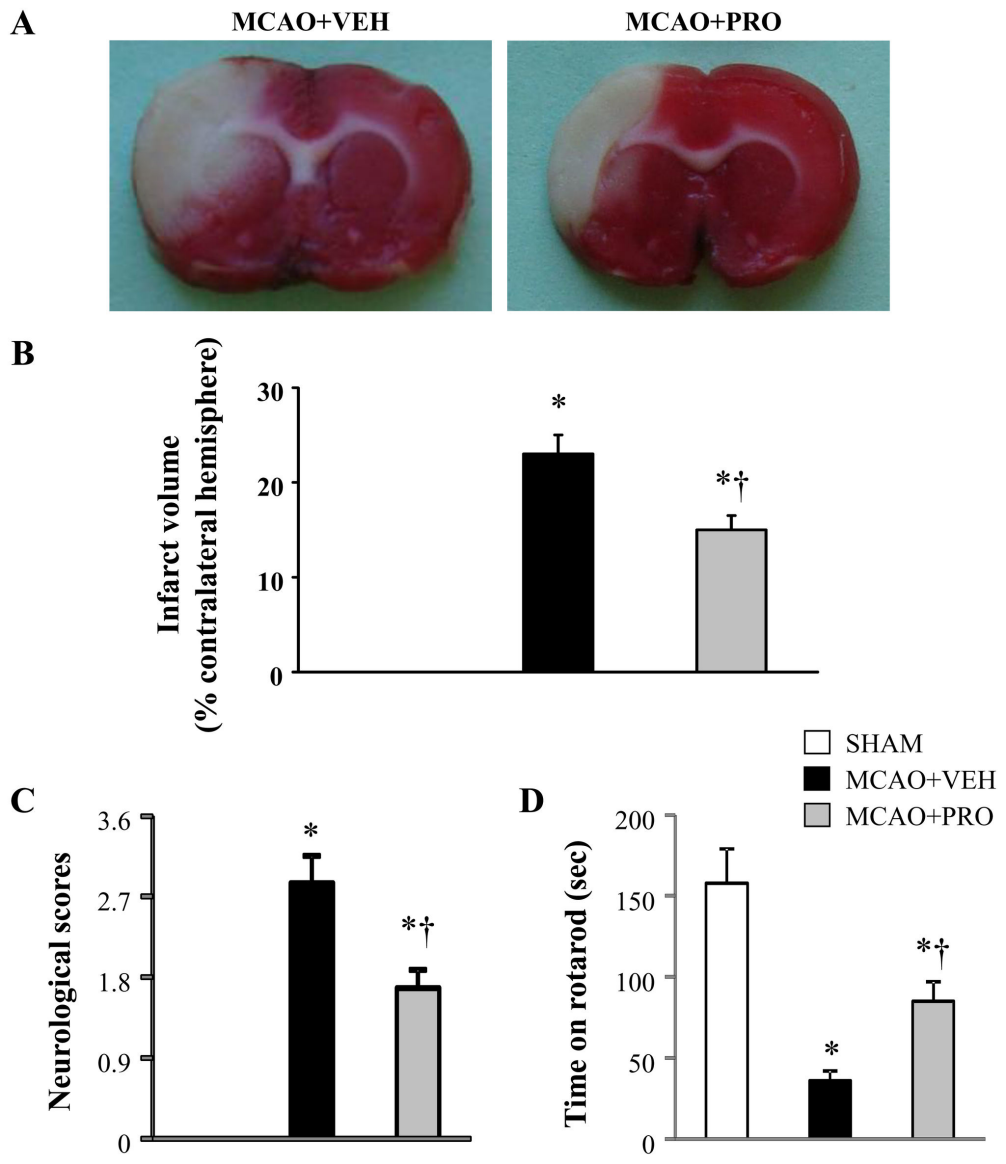
Representative coronal brain slices stained with TTC (A), infarct volume (B), neurological deficit scores (C) and motor coordination (D) 24 h after middle cerebral artery occlusion (MCAO) in rats treated with vehicle (VEH) or propofol (PRO). Sham-operated rats (SHAM) without treatment were used as control. Values are mean ± SEM (n = 8 for each group). *P< 0.05 vs. SHAM, †*P*< 0.05 MCAO+PRO vs. MCAO+VEH.

A 2-h MCAO followed by 24 h reperfusion induced an infarct volume of 23 ± 2% in vehicle-treated rats (Figure **1A and 1B**). In contrast, treatment with propofol after MCAO reduced infarct volume significantly by approximately 35% (p < 0.05). 

The time spent on rotarod among three groups was similar before MCAO (average time on rotarod was 154 ± 17 sec). Twenty-four hours after MCAO, vehicle-treated rats exhibited markedly higher neurological deficit scores (Figure **1C**) and reduced time on rotarod (Figure **1D**) than sham rats. Whereas rats treated with propofol after MCAO demonstrated significant decrease in neurological deficit scores and improvement in rotarod performance compared with vehicle-treated rats. 

### Propofol inhibited MCAO-induced microglial activation

**Figure 2:**
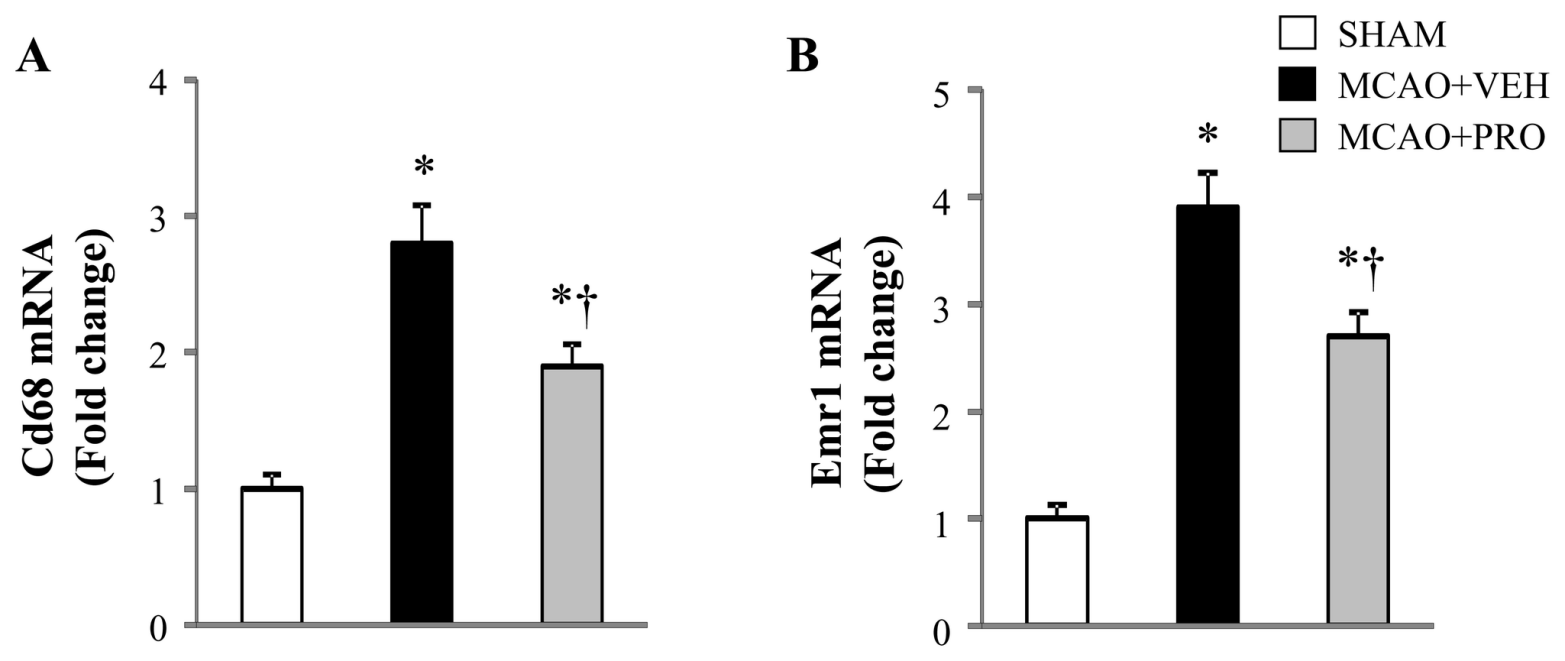
mRNA expression for microglia specific markers Cd68 (A) and Emr1 (B) in the peri-infarct cortical tissue 24 h after MCAO in rats treated with VEH or PRO. SHAM rats without treatment were used as control. Values are mean ± SEM (n = 8 for each group) and expressed as a fold change relative to SHAM. *P< 0.05 vs. SHAM, †*P*< 0.05 MCAO+PRO vs. MCAO+VEH.

Real-time PCR showed that mRNA expression of Cd68 and Emr1, two microglia specific markers, markedly increased by 178% and 290%, respectively, in the peri-infarct cortical tissue in vehicle-treated rats 24 h after MCAO as compared to those in sham rats (Figure **2**). Compared with vehicle-treated rats, propofol-treated rats had significantly decreased mRNA expression of Cd68 and Emr1 in the peri-infarct cortical tissue 24 h after MCAO. 

**Figure 3:**
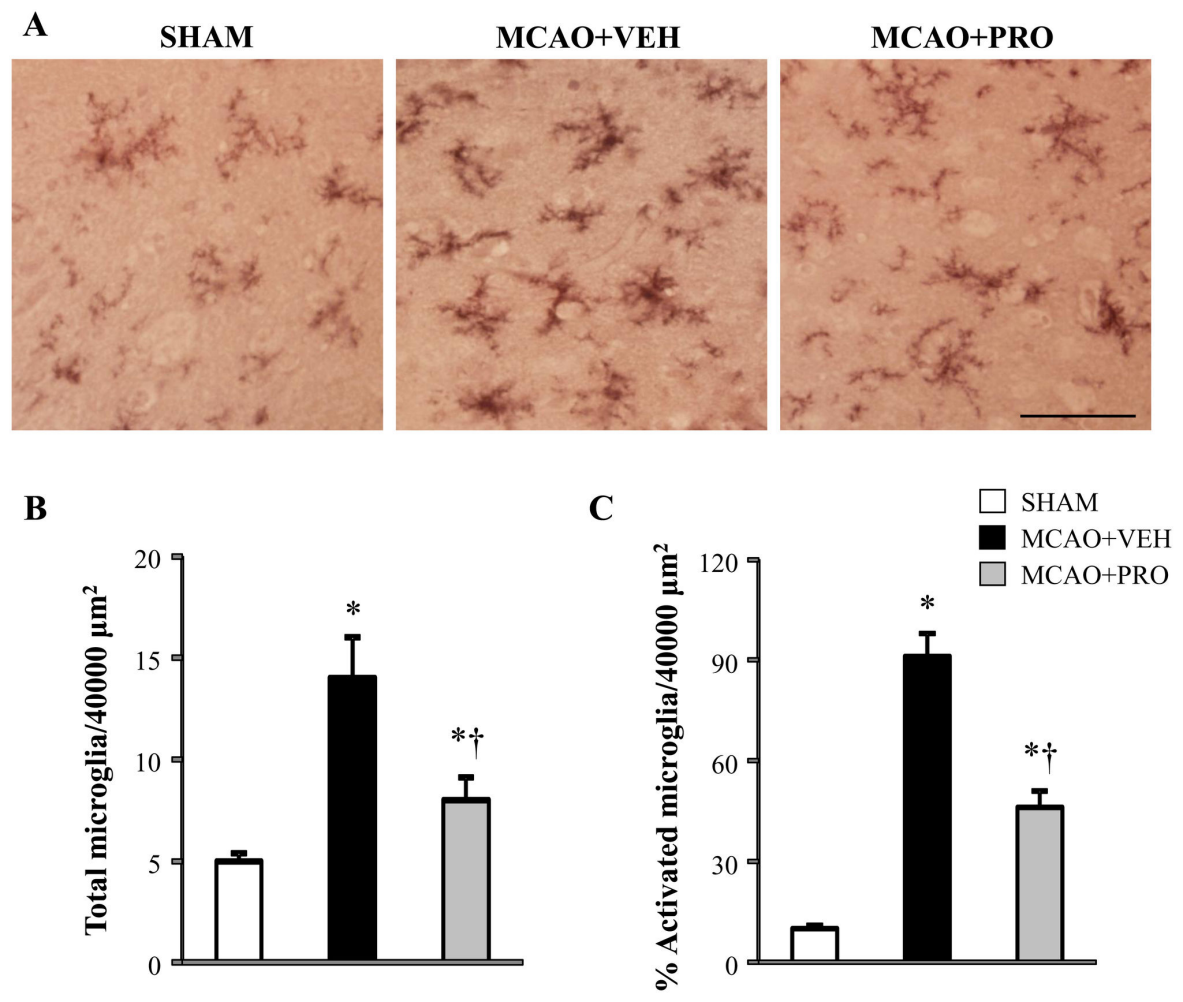
Representative photomicrographs of microglia stained with CD11b (A), total number of microglia (B) and the proportion of activated microglia (expressed as percent of total microglia) (C) in the peri-infarct cortical tissue 24 h after MCAO in rats treated with VEH or PRO. SHAM rats without treatment were used as control. Activated microglia were defined as strong CD11b immunoreactivity, an enlarged soma, and fewer and shorter processes. Scale bar, 200 μm. Values are mean ± SEM (n = 4 for each group). *P< 0.05 vs. SHAM, †*P*< 0.05 MCAO+PRO vs. MCAO+VEH.

OX42 antibody is a specific microglial marker and stains all microglia. Activated microglia were defined as cells that exhibit strong OX-42 immunoreactivity, an enlarged soma, fewer and shorter processes. Using immunohistochemical study, we found that microglia were presented in the cortical tissue of sham rats, but there were few microglia with an activated morphology (Figure **3A**). The average number of total microglia (Figure **3B**) and the proportion of activated microglia (Figure **3C**) counted in the peri-infarct cortical tissue were significantly increased in vehicle-treated rats 24 h after MCAO compared with those in sham rats. In contrast, both the number of total microglia and the proportion of activated microglia in the peri-infarct cortical tissue were reduced in rats treated with propofol.

### Propofol attenuated MCAO-induced proinflammatory cytokines

**Figure 4:**
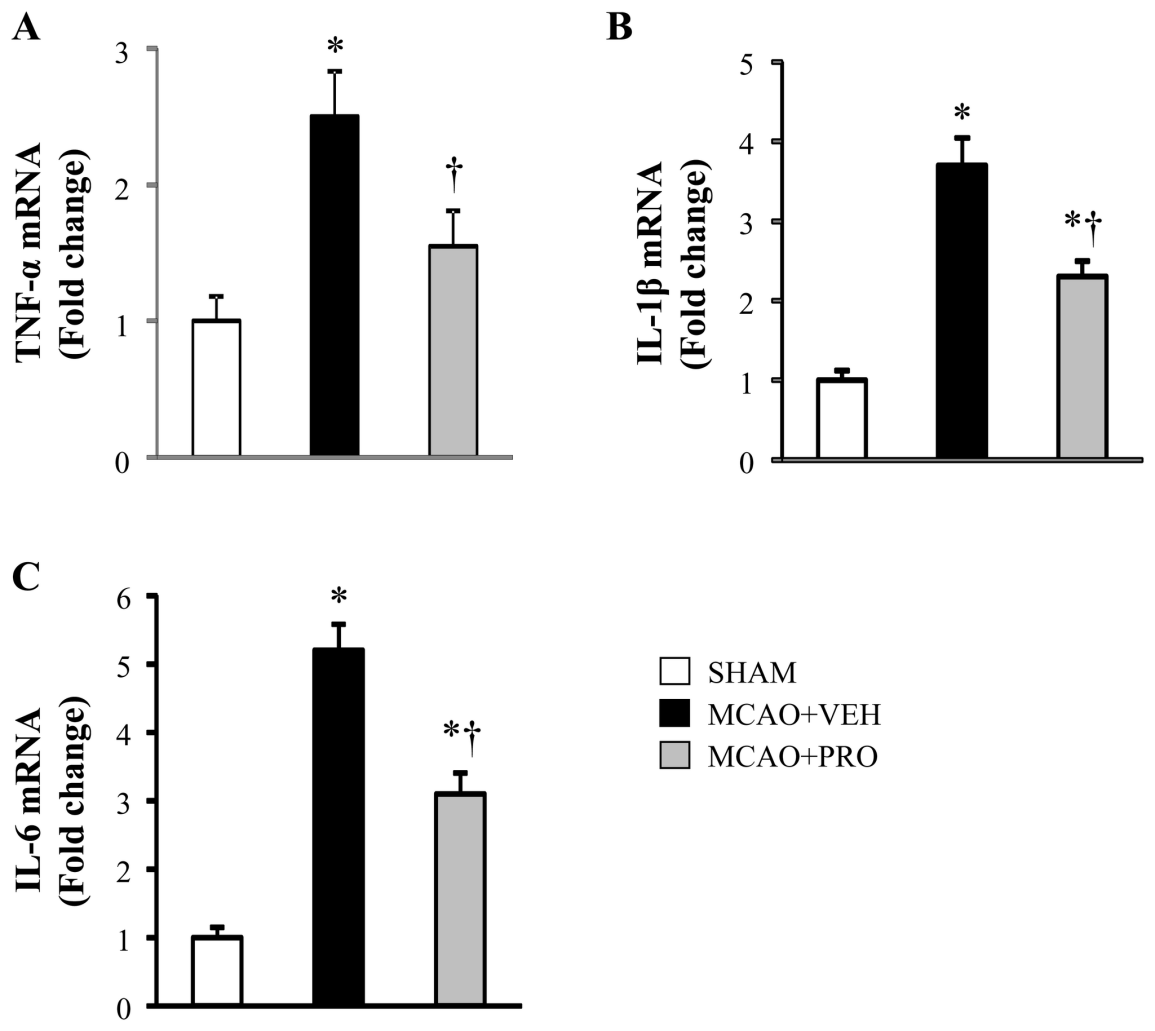
mRNA expression for proinflammatory cytokines TNF-α (A), IL-1β (B) and IL-6 (C) in the peri-infarct cortical tissue 24 h after MCAO in rats treated with VEH or PRO. SHAM rats without treatment were used as control. Values are mean ± SEM (n = 8 for each group) and expressed as a fold change relative to SHAM. *P< 0.05 vs. SHAM, †*P*< 0.05 MCAO+PRO vs. MCAO+VEH.

**Figure 5:**
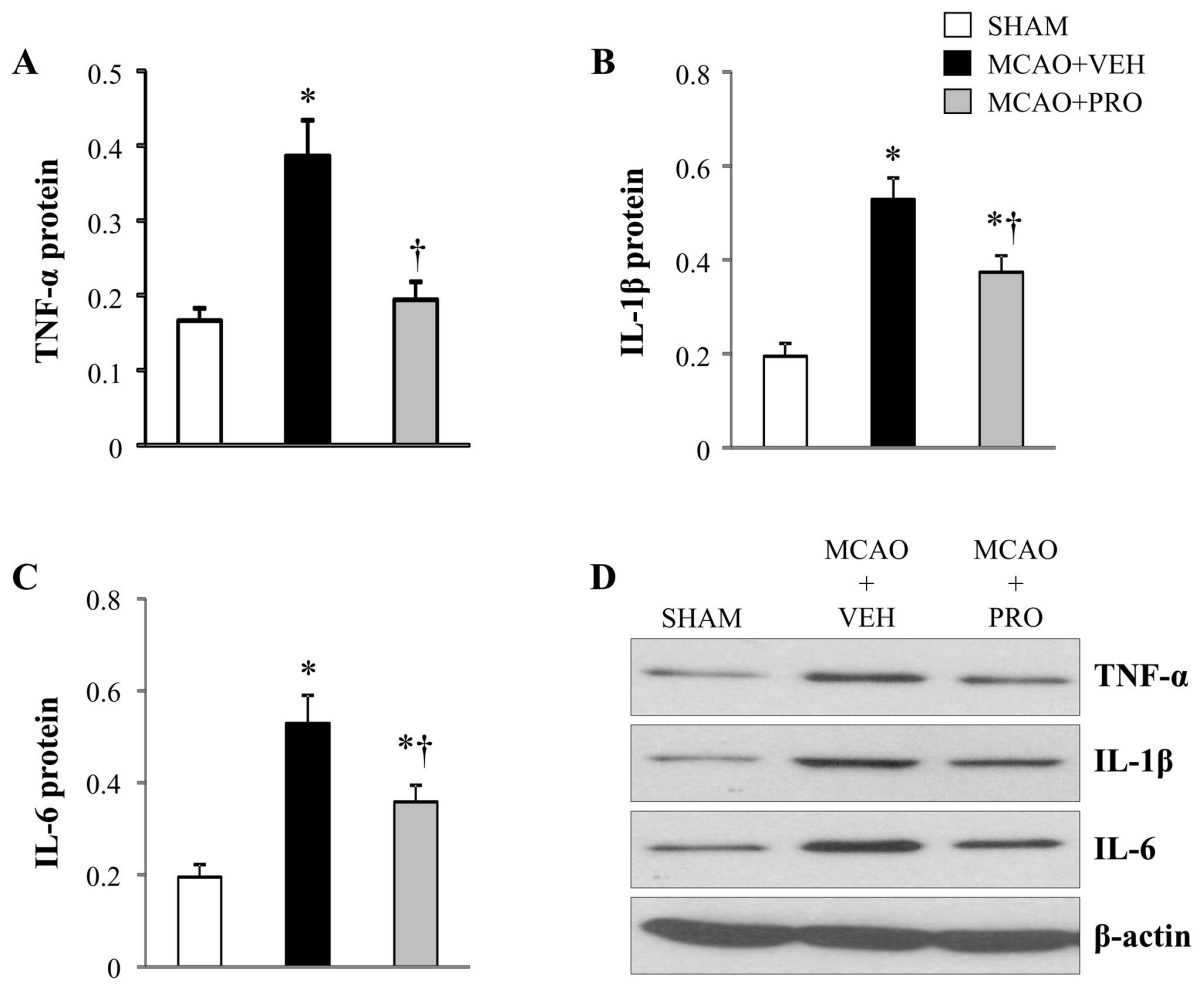
Protein levels for proinflammatory cytokines TNF-α (A), IL-1β (B) and IL-6 (C) in the peri-infarct cortical tissue 24 h after MCAO in rats treated with VEH or PRO. SHAM rats without treatment were used as control. Representative Western blots are shown in figure D. Values are expressed as mean ± SEM (n= 8 for each group) and corrected by β-actin. *P< 0.05 vs. SHAM, †*P*< 0.05 MCAO+PRO vs. MCAO+VEH.

Multiple proinflammatory cytokines play an important role in the regulation of inflammation. TNF-α, IL-1β and IL-6 are major early response cytokines that trigger a cascade of inflammatory mediators, including other cytokines, chemokines, reactive nitrogen or oxygen intermediates [23]. In the brain, microglia produce all 3 cytokines. C-reactive protein is an exquisitely sensitive systemic marker of inflammation and tissue damage. In the present study, the neuroprotective actions of propofol administrated after MCAO could be due to its inhibitory effects on microglia and subsequent production of proinflammatory cytokines in the brain and periphery. To test this hypothesis, we measured the levels of above proinflammatory cytokines in the brain and plasma. The mRNA (Figure **4**) and protein (Figure **5**) expressions of the proinflammatory cytokines TNF-α, IL-1β and IL-6 were significantly augmented in the peri-infarct cortical tissue of vehicle-treated rats compared with sham rats. After treatment with propofol, mRNA and protein expressions of IL-1β and IL-6 were significantly reduced, and mRNA and protein expressions of TNF-α were normalized in the peri-infarct cortical tissue of rats at 24 h following MCAO.

**Figure 6:**
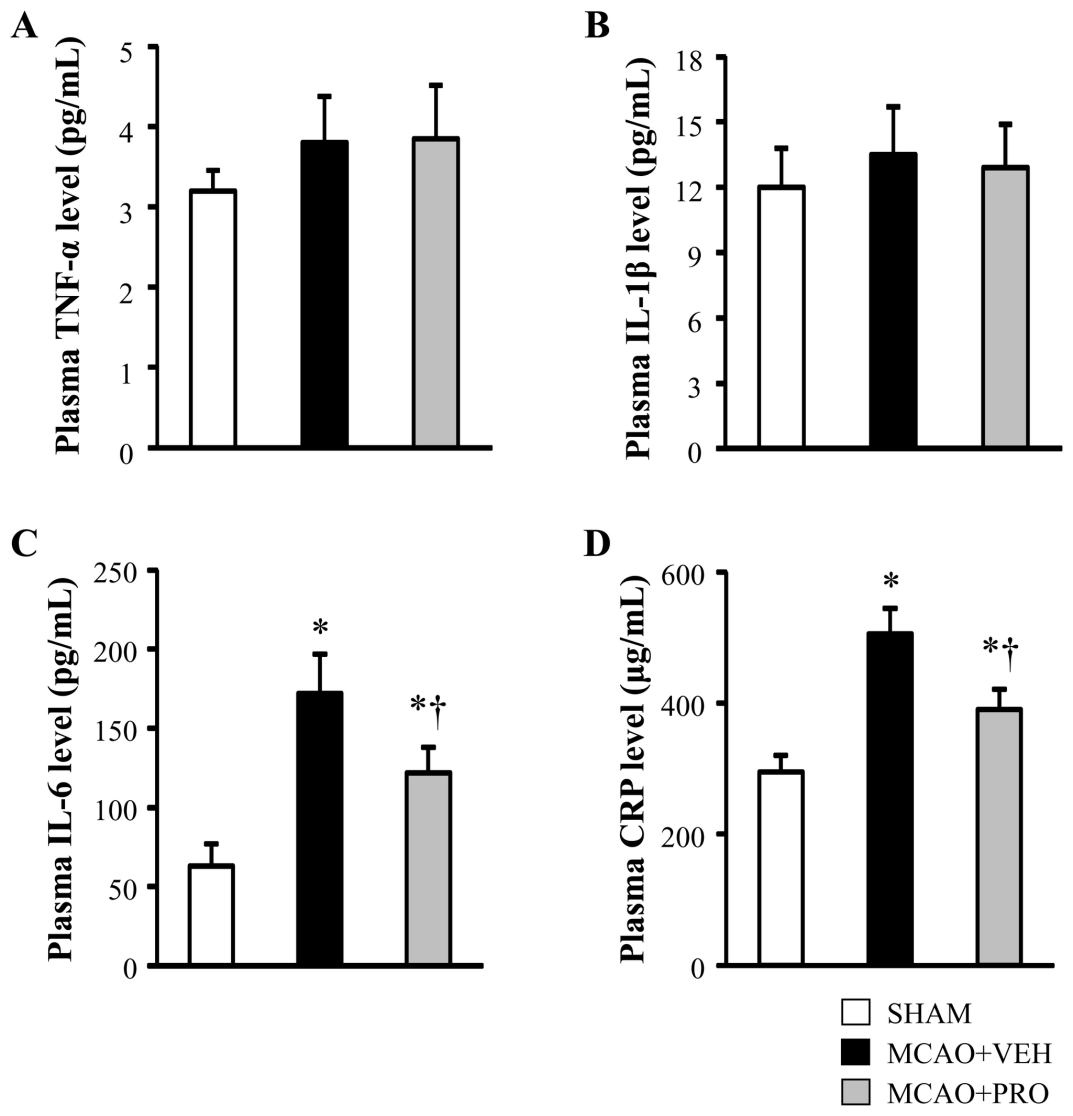
Plasma levels of proinflammatory cytokines TNF-α (A), IL-1β (B), IL-6 (C) and C-reactive protein (D) 24 h after MCAO in rats treated with VEH or PRO. SHAM rats without treatment were used as control. Values are mean ± SEM (n = 8 for each group). *P< 0.05 vs. SHAM, †*P*< 0.05 MCAO+PRO vs. MCAO+VEH.

There were no differences in plasma levels of TNF-α and IL-1β across the 3 experimental groups (Figure **6A and 6B**). However, vehicle-treated rats had higher plasma levels of IL-6 and C-reactive protein, which were significantly reduced after treatment with propofol (Figure **6C and 6D**).

## Discussion

The novel finding of this study is that treatment with propofol early after ischemic stroke suppressed microglia activation and proliferation in the peri-infarct cortical regions, reduced the production of proinflammatory cytokines in the brain as well as in peripheral blood, and improved neurological outcome. To our knowledge, this is the first study *in vivo* to demonstrate that the propofol confers neuroprotection against ischemic brain injury by modulating microglial function. Our finding provides new understanding of the protective mechanisms of anesthetic propofol, which may be applicable in the immediate aftermath of stroke as well as to patients with a stroke history undergoing surgery, patients in the intensive care unit under sedation, and patients undergoing neurosurgery. 

Propofol has become the most widely used anesthetics in neurosurgery. More recently, the anti-inflammatory functions of propofol have been received much attention because this agent has been shown to exert protective effects during acute inflammatory in neurologic and cardiovascular diseases [10,24]. For example, experimental studies in animals showed that propofol inhibits cytokine release during sepsis [25,26] and decreased neutrophil-mediated inflammation in acute pulmonary injury [27]. Clinical studies revealed that propofol attenuates myocardial reperfusion injury and pulmonary dysfunction following cardiopulmonary bypass by reducing free radical release and modulating the inflammatory process [28,29]. In addition, a number of studies have reported that propofol protects against ischemic brain injury in animal models [12-14], but the underlying mechanisms remain unclear. In the present study, we found that a 2-h MCAO followed by 24 h reperfusion elicited large brain infarct in the frontoparietal cortex. Administration of propofol early after MCAO reduced infarct volume, improved neurological outcome as evidenced by decrease neurological deficit scores and increased time in rotarod performance. These results are consistent with previous studies, suggesting a protective effect of propofol on ischemic brain injury. More importantly, our data extend previous findings by revealing that the beneficial effects of propofol on ischemic brain injury are associated with suppression of microglial activation and proliferation in the peri-infarct cortical areas and reduction in releasing proinflammatory cytokines. 

The inflammatory responses in the brain to ischemic stroke are characterized by a rapid activation and proliferation of microglial cells, followed by the infiltration of circulating inflammatory cells, including neutrophils, T cells, monocyte/macrophages, and other cells in the ischemic brain region, as demonstrated in animal models and in stroke patients [4,30,31]. The microglia are activated within minutes after onset of focal cerebral ischemia and may last for several weeks after initial injury [4]. Activated microglia produce a plethora of proinflammatory mediators in the brain, including TNF-α, IL-1β and IL-6, which contribute to the expansion of brain injury and the delayed loss of neurons [3,7-9,32]. It has been shown that intraventricular injection of IL-1 and TNF-α increases infarct volume and brain edema after MCAO in rats, whereas the injection of microglial inhibitor minocycline [33,34] or PPAR**-**γ agonist pioglitazone that suppresses microglial activation and expression of proinflammatory cytokines [35], or administration of antibodies against IL-1 and TNF-α [8,36] reduces brain injury. The data of present study showed that, at 24 h after MCAO, mRNA expression of microglial markers Cd68 and Emr1 in the peri-infarct cortical tissue was augmented, and the number of total microglia and the proportion of activated microglia were increased, suggesting that ischemic stroke results in microglial activation and proliferation in this brain area. In addition, expression of proinflammatory cytokines TNF-α, IL-1β and IL-6 in this brain area was also increased. These results are consistent with previous findings showing that cerebral ischemia substantially activates microglia and increases expression of proinflammatory cytokines in the frontoparietal cortex adjacent to the ischemic core 24 h after MCAO, and increased immunoreactivity for proinflammatory cytokines is mostly co-localized with activated microglia [21,37], indicating that activated microglial cells are the main source of proinflammatory cytokines in the brain after ischemic stroke. Furthermore, we found that early treatment with propofol reduced mRNA expression of Cd68 and Emr1, decreased the number of total microglia and the proportion of activated microglia in the peri-infarct cortical tissue, accompanied by decreased mRNA and protein expressions of proinflammatory cytokines 24 h after MCAO. These findings provided evidence for suppressive effects of propofol on microglial activation and release of proinflammatory cytokines *in vivo* in rats after ischemic stroke. Our current data are supported by recent *in vitro* studies showing that propofol dramatically reduced levels of proinflammatory cytokines TNF-α, IL-1β and IL-6, and activation of microglia induced by lipopolysaccharide [11,38] or extracellular pressure [39]. Taken together, these observations demonstrate that the beneficial effects of propofol on infarct volume and neurological outcome are associated with inhibition of microglia activation and suppression of the exaggerated production of proinflammatory cytokines in ischemic brain early after ischemic stroke. 

The use of biochemical markers as predictors of stroke lesion evolution and prognosis is becoming increasingly important, as they may be valuable tools in the search for an optimal management of stroke patients. It is notable that cerebral ischemia did not change the levels of plasma proinflammatory cytokines TNF-α and IL-1β, but significantly increased the levels of plasma IL-6 and C-reactive protein, which were attenuated by propofol treatment. Inflammation in the brain is known to modulate inflammation in the periphery in ischemic stroke, and measurement of peripheral inflammatory response has been suggested to be a far more practical approach in clinical research [40]. Elevations of proinflammatory cytokines and C-reactive protein in plasma after ischemic stroke have been reported in both clinical and experimental studies [3,41-43]. The acute-phase response, characterized by elevated plasma concentrations of IL-6, C-reactive protein and neutrophil leukocytosis, is induced within hours of ischemic stroke [42]. Parameters of the acute-phase response, particularly plasma IL-6 and C-reactive protein concentrations, are positively associated with stroke severity and infarct volume, and predict a higher risk of early clinical worsening [3,40].Thus, reductions in plasma proinflammatory cytokines IL-6 and C-reactive protein after treatment with propofol in our study are likely to reflect decreased risk of early clinical deterioration. 

Two major limitation of the present study should be acknowledged. First, it has been reported that cerebral ischemia also induces activation of astrocytes, another resident cells in the brain, which can produce the proinflammatory cytokines including TNF-α, IL-1β and IL-6. Intervention to inhibit astrocyte activation has been shown to enhance neuronal survival and improve outcome following cerebral ischemia [44,45]. However, the present study focused only on the role of microglia activation in ischemic brain injury, further studies are needed to determine whether neuroprotective effects of propofol observed in this study are partially due to inhibition of astrocyte activation. Second, although propofol treatment suppressed microglia activation and reduced the production of proinflammatory cytokines in the brain as well as in peripheral blood, accompanied by decreased infarct size and improved neurological outcome, but the finding of decreases in these inflammatory markers does not prove that these cause the decrease in infarct size. Other important mediators associated with ischemic neuronal injury might also be reduced by propofol and contributed to the decrease in infarct size. Further research is necessary to determine whether there is a direct relationship between the levels of these proinflammatory cytokines and infarct size after ischemic stroke.

In conclusion, the present study demonstrates that administration of propofol early after cerebral ischemia reduces infarct volume and improves neurological function by inhibition of microglia activation and proinflammatory cytokine release in the brain. Propofol may be a promising therapeutic agent for the prevention and/or treatment of ischemic brain injury and other neurodegenerative diseases associated with microglial activation. 
